# Preliminary study of cyclosporine A/Lifitegrast subconjunctival sustained-release drug membrane in the treatment of dry eyes

**DOI:** 10.1186/s40662-024-00390-5

**Published:** 2024-06-13

**Authors:** Jie Yang, Miao Chen, Fangyuan Wu, Jingjing Zuo, Huixiang Ma

**Affiliations:** 1 National Clinical Research Center for Ocular Diseases, Eye Hospital, Wenzhou Medical University, Wenzhou, 325027 China; 2 State Key Laboratory of Ophthalmology, Optometry and Vision Science, Eye Hospital, Wenzhou Medical University, Wenzhou, 325027 China

**Keywords:** Cyclosporine A, Lifitegrast, Subconjunctival membrane implantation, Sustained release membrane, Rabbit dry eye model

## Abstract

**Background:**

Dry eyes can cause discomfort. To treat dry eye disease, cyclosporine A (CsA) and Lifitegrast are two eye drugs approved by the U.S. Food and Drug Administration (FDA). However, frequent use of eye drops can be challenging and lead to poor compliance, especially in elderly patients. Therefore, this study aimed to develop a drug sustained-release vector and explore its therapeutic effect in animal models of dry eye.

**Methods:**

Firstly, drug membranes loaded with both CsA and Lifitegrast using a carrier called poly(lactate-co-ε-caprolactone) (P(LLA-CL)) were prepared and evaluated for their physicochemical properties, release behavior in vitro, and safety in vivo. Next, a rabbit dry eye model using a 0.1% benzalkonium chloride (BAC) solution was developed and treated by drug-loaded micro membranes. We observed and recorded conjunctival hyperemia, corneal staining, corneal edema, corneal neovascularization, conjunctival goblet cells and hematoxylin and eosin (H&E) staining. Finally, we detected the MUC5AC and MMP-9 by immunofluorescence staining and enzyme-linked immunosorbent assay (ELISA).

**Results:**

The composite film released both CsA and Lifitegrast for at least one month. Compared to the blank membrane group, conjunctival hyperemia, corneal fluorescein staining, corneal edema, corneal neovascularization and conjunctival goblet cells recovered faster in the drug membrane group, and the difference was statistically significant. At the molecular level, the drug membrane group showed an increase in mucin density and a significant anti-inflammatory effect.

**Conclusions:**

The implantation of CsA/Lifitegrast loaded P(LLA-CL) membrane under the subconjunctival of the rabbit eye is safe. The study suggests that this subconjunctival administration could be developed into a minimally invasive delivery system to help patients with dry eye disease who require multiple daily eyedrops but have poor compliance.

## Background

In 2017, the Tear Film and Ocular Surface Society’s Dry Eye Workshop II (TFOS DEWS II) revised the definition of dry eye, stating that dry eye is now a multifactorial chronic ocular surface disease. Major pathophysiological mechanisms such as tear film instability, hyperosmolarity of tears, epithelial cell apoptosis, and inflammation are interconnected, forming a vicious cycle giving rise to dry eye [1]. The inflammation mediated by T cells and related cytokines is considered the core driving factor of dry eye disease (DED) [2, 3]. In this immunomodulatory process, the increase of tear osmolalities can activate the MAPK pathway [2]. This pathway activates the master regulator NFkB, producing inflammatory factors such as interleukin (IL)-1 (major), IL-17, tumor necrosis factor (TNF)-α, interferon (IFN)-γ, and matrix metalloproteases (MMPs). It also induces other mediators and cell signal cascades, which amplify the inflammatory immune response. This can result in T cell differentiation, proliferation, and recruitment. Consequently, there can be an infiltration in conjunctival corneal tissue, causing inflammation that affects goblet cells, epithelial cell apoptosis, mucin, tear film instability, and tear osmolarity. These reactions further promote the inflammatory response [4]. Throughout inflammation, the acute-phase cytokines IL-1 and TNF-α induce increased ICAM-1 expression on a variety of cells, whose interaction with LFA-1 is thought to be required for the activation of ocular surface effector T cells through the immunological synapse. Their interaction also plays a role in the recruitment of the conjunctival epithelium and ocular surface T cells [5].

At present, there are many therapeutic drugs for DED including various artificial tear, aqueous and mucous secretion stimulants (such as P2Y_2_ receptor agonist, diquafosol sodium, etc.), immunosuppressants, and glucocorticoid eye drops. As approved by the U.S. Food and Drug Administration (FDA) for topical treatment of DED at 2002, 0.05% cyclosporin A (CsA) has been shown in many retrospective clinical analyses to improve dry eye MMP-9 levels, Schirmer score, corneal staining and breakup time of tear film. Many studies have reported that CsA plays a role in DED immunopathophysiology, including inhibiting T cell activation, reducing cyclophilin, mediating IL-2 and IL-6 gene transcription, reducing epithelial and goblet cell apoptosis, and increasing tear production and conjunctival goblet cell density in patients with keratoconjunctivitis [6]. Lifitegrast (5%), approved by the FDA in July 2016 to treat DED, is a new drug that belongs to the class of lymphocyte function-related antigen-1 (LFA-1) antagonists. It can block the interaction between the cell surface protein LFA-1 and inter-cell adhesion molecule-1 (ICAM-1), and thus impede the inflammatory cascade associated with DED by inhibiting the migration of activated T cells to the conjunctiva and recruitment in the conjunctival epithelium and secondary activation in the eye tissue [7]. The drug showed significant improvement of symptoms in patients with dry eyes, and no safety problems were found with long-term use. However, safety assessment of the drug reported that a few patients using 5% Lifitegrast showed irritation and were in pain, although the discomfort would disappear within 3 min [8]. Although CsA and Lifitegrast have proven to be very effective in the treatment of dry eyes, intolerance to these topical drops has been an important barrier to treatment. These drops cause burning, pain, and irritation at the infusion site to greatly reduce patient compliance. There is a need to improve the dosage of such drugs to reduce drug irritation.

Currently, eye drops are the most used treatment for acute eye diseases. Although they are convenient, they require multiple applications throughout the day due to the quick removal of the drug, resulting in a significant amount of drug waste. This is especially true for CsA and Lifitegrast eye drops, which can greatly increase the economic burden on patients. Additionally, patients who require multiple eye drops often struggle with poor compliance. To maintain an effective therapeutic concentration for several hours, the actual concentration used is often much higher, resulting in side effects such as stimulating symptoms that hinder subsequent treatment. Subconjunctival injection, which has a higher biological utilization rate than eye drops, is commonly used clinically due to the large conjunctival surface area and good permeability [9]. The adoption of subconjunctival drug delivery systems with sustained-release capabilities can significantly reduce drug surface stimulation, improve drug utilization, and release the drug in a safe and effective concentration. This can reduce the need for multiple daily eye drops, bringing convenience and improved quality of life for patients.

PolyL(L-lactide)-co-poly(ɛ-caprolactone), also known as P(LLA-CL), is a copolymer of FDA-approved biomaterials PolyL-propylene(Poly(L-lactide)) and polycaprolactone(Poly(ɛ-caprolactone)). This copolymer has been successfully used for sustained release of ocular drugs and has good compatibility with animal eyes [10]. Common drug delivery forms used in ophthalmology include nanoparticles, liposomes, nanoemulsions, and drug-loaded membranes. Compared to the former, the advantage of drug-loaded membranes lies in their drug-loading efficiency being unaffected by the solubility of the drug (whether water-soluble or lipid-soluble), allowing for a significant increase in drug loading. Additionally, by controlling the degradation rate of the microfilm carrier, the drug release rate can be adjusted, thereby effectively improving drug bioavailability. Our hypothesis is that if P(LLA-CL) is made into a micron-thick diaphragm for subconjunctival implantation, it can be used for sustained release administration of CsA and Lifitegrast. The size of the implant and the duration of drug release determine the invasiveness of subconjunctival implants. Minimally invasive 1–2 mm subconjunctiva is much safer than intravitreal injection (currently widely used). Our study aims to examine the feasibility of subconjunctival implantation of this composite drug membrane and evaluate its sustained-release efficacy and pharmacodynamics on a rabbit dry eye model. To facilitate manipulation and drug quantification, a relatively large diameter diaphragm (5 mm diameter) was selected. The thickness of the diaphragm is controlled at the micron level (160–170 μm ) to reduce foreign body sensation. Benzalkonium chloride (BAC) is a commonly used preservative in ophthalmic preparations. It has been shown to cause tear film instability, loss of conjunctival goblet cells, reduction of mucin (e.g., MUC5AC), conjunctival squamous metaplasia and apoptosis, corneal epithelial barrier destruction and deep ocular tissue damage. Studies have demonstrated that 0.1% BAC solution can induce a more stable dry eye model (DED) on rabbit eyes [11]. Therefore, we chose a stable rabbit dry eye model induced by 0.1% BAC solution to evaluate the therapeutic effect of the drug membrane. Matrix metalloproteinase-9 (MMP-9) [12–15] is the only FDA-approved biomarker for the diagnosis of dry eyes and provides objective value for the care and clinical studies of DED patients. Many studies have also shown that MMP-9 is higher in tears in DED. Therefore, we examined the concentration of MMP-9 in tears to evaluate the effectiveness of the drug membrane.

## Methods

### Animals

Normal healthy adult Japanese White Rabbit was provided by the Laboratory Animal Center of Wenzhou Medical University (SYXK (Zhejiang) 2014-0006). All animals conformed to the experimental standard (SCXK (Zhejiang) 2013-0057) of both sexes, with no eye diseases after screening, and weighed between 2.0 and 2.5 kg on average.

### Materials

P(LLA-CL) (50/50, IV: 1.0 dL/g, Mn 70,000–90,000) was purchased from Jinan Daigang Biological Engineering Co., Ltd. Drug compounds used were Lifitegrast and CsA (Sigma, USA). The organic solvent used were acetone and dichloromethane (analytical pure, Sigma, USA), methanol and acetonitrile (HPLC grade, Merck, Germany). All other chemicals were analytically pure without further purification. MUC5AC Mouse polyclonal antibody (Thermo Fisher Scientific), FITC-conjugated Affinipure Goat Anti-Mouse IgG (H + L) (Proteintech Group), and the MMP-9 enzyme-linked immunosorbent assay (ELISA) kit was purchased from RayBiotech.

### Preparation of the drug film

P(LLA-CL) and CsA (in a mass ratio of 2:3) were dissolved in dichloromethane. Separately, P(LLA-CL) and ricastin were dissolved in acetone in a 2:3 mass ratio to create a P(LLA-CL)-listrast-acetone solution. CsA and Lifitegrast were used in equal parts and the two solutions had equal volumes. To create a CsA/Lifitegrast composite film, the Teflon plate was placed on a heating plate at 60℃. The nitrogen tank pressure valve was adjusted to 0.1 MPa, and a spray gun (W77-G, Iwata Japan) was used to alternately spray 2mL of P(LLA-CL)-CsA solution and 2mL of P(LLA-CL)-Lifitegrast solution to achieve a composite membrane/film with a 30% load and a thickness of 150–170 μm.

### Determination of drug membrane thickness and drug loading

A number of circular samples were drilled on the resulting drug film (30 × 50 mm) using a corneal trephine(φ 5 mm). The thickness of the drug film was measured with a spiral micron micrometer. The samples were weighed and dissolved in 5mL acetonitrile. After complete dissolution, the supernatant was centrifuged, and the drug loading of the two drugs on the drug film was measured by high-performance liquid chromatography (HPLC).

### Safety profile of the membrane

Drug-loaded film and blank film (both with a diameter of 5 mm) were sterilized using ultraviolet (UV) radiation for 20 min. Six healthy Japanese white rabbits of both sexes were randomly selected. After anesthesia and disinfection, a circular drug film was implanted at 10:30 under the conjunctiva, positioned 3 mm behind the corneoscleral margin. The conjunctival incision was closed using an absorbable 8 − 0 suture. Following the surgery, the eyes were rinsed with normal saline solution and ofloxacin eye ointment was instilled into the conjunctival sac. To prevent infection, the ofloxacin eye ointment was applied three times daily for three consecutive days after surgery. Conjunctival hyperemia, anterior chamber flare, anterior chamber cells, corneal fluorescein sodium staining, and Rose Bengal staining were evaluated on Days 3, 7, 14, 21, and 28 post-implantation. Conjunctival impression cytology (CIC) was performed weekly to assess goblet cell count and weekly intraocular pressure monitoring was also performed. From Weeks 1 to 6 following drug film implantation, photographs of the implant site were taken every week under an operating microscope to observe any adverse reactions related to foreign body implantation. At the end of Week 6, animals were sacrificed, and their eyes removed for pathological examination in order to observe inflammatory cells surrounding the graft.

### Drug film characterization

#### Scanning electron microscopy (SEM)

CsA/Lifitegrast composite film (upper and lower sides), Lifitegrast alone, and CsA alone, were imaged under the scanning electron microscope to observe the loading membrane and drug microstructure.

#### Fourier-transform infrared spectroscopy (FTIR)

The sample to be tested and pure potassium bromide were evenly mixed at a ratio of 1:99, thoroughly mixed and dried. The samples tested were a composite membrane loaded with CsA/Lifitegrast, a blank membrane of P(LLA-CL), CsA naked drug, Lifitegrast naked drug, and a mixture of CsA and Lifitegrast naked drugs. The drug and blank films containing the polymeric material P(LLA-CL) were dissolved in dichloromethane, mixed well, and dried at 54 °C. The resulting solid mixture was ground into a uniform powder using an agate mortar, and then the powder was made into thin flakes using a pressing machine at a pressure of 0.15 KPa. The thin slices were then measured using a Fourier transform infrared spectrometer after scanning the background spectra. The scan range was 400 to 4000 cm^−1^, with a resolution of 4 cm^−1^ and 32 scans. The final reading is presented as % transmittivity.

#### Differential scanning thermal method (DSC)

Appropriate amounts of CsA/Lifitegrast composite drug film, P(LLA-CL) blank film, active drug Lifitegrast and CsA were weighed into an aluminum dish, covered, and pressed into thin slices by a tablet press. The furnace was opened at room temperature, and the sample and reference were carefully and smoothly placed into the calorimeter of the differential scanning calorimeter with forceps. The glass transition temperatures of the drug film, blank film and the two bulk drugs were measured by heating the samples from − 80 °C to 220 °C, 280 °C, 250 °C and 260 °C, respectively at a rate of 10 °C per minute in a nitrogen atmosphere.

#### Drug membrane release in vitro

All the drug films were circular with a diameter of 5 mm. Six tablets of CsA/Lifitegrast composite drug films (*n* = 6), and three tablets of free CsA/Lifitegrast mixed naked drug films with an equal amount (*n* = 3) were used. Three tablets of composite drug films (*n* = 3) and naked drug films (*n* = 3) were put into the dialysis bag (truncated molecular weight 14,000, Viskase, USA). The opening of the dialysis bag was fastened with thread and then immersed into a 15 mL polypropylene tube containing 5mL 0.01 M phosphate-buffered saline (PBS) solution. The remaining composite drug films (*n* = 3) were separately immersed into 15 mL polypropylene tubes containing 5 mL 0.01 M PBS solution. The tubes were placed on a 37 °C 100 rpm thermostatic shaker (model 481, Themo, USA). The release solution was collected and replaced daily. Drug concentrations were measured by ultra-performance liquid chromatography (UPLC). The in vitro release was stopped when the drug concentration remained undetectable for a week.

### Pharmacodynamic study of the drug membrane

**Fig. 1:**
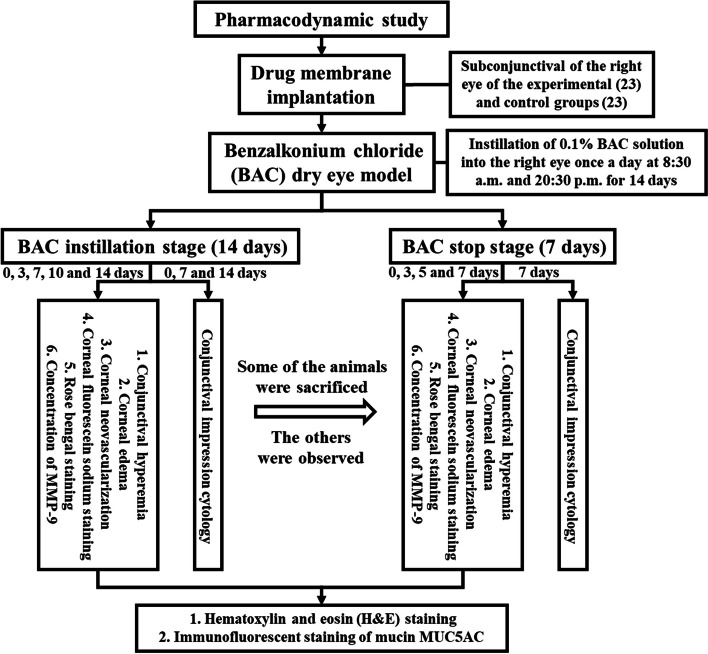
Flow-chart showing the pharmacodynamic study of the drug membrane

Figure 1 illustrates the pharmacodynamic study design. Firstly, the UV-sterilized CsA/Lifitegrast and P(LLA-CL) blank membrane circles were implanted into the subconjunctiva of the right eye of the experimental and control groups as described above. Two weeks after implantation, the BAC dry eye model was induced by instillation of 0.1% BAC solution into the right eye (the left eye served as the control and received saline solution) once a day at 8:30 a.m. and 20:30 p.m. Conjunctival hyperemia, corneal edema, corneal neovascularization, corneal fluorescein sodium staining and Rose Bengal staining were recorded at 0, 3, 7, 10 and 14 days after first BAC induction. CIC was performed at 0, 7 and 14 days after first BAC induction. If there was severe neovascularization, lamellar staining, corneal ulcer or even perforation tendency in the right eye, the medication was stopped, the withdrawal time was recorded, and CIC was performed on the same day. If there was no indication for withdrawal, CIC was performed on the same day. Some of the animals in both groups were sacrificed on the same day, and the others were observed for another week to record the recovery period. All rabbit eyes at the BAC stage were similarly examined and recorded above on the 3rd, 5th and 7th day after stopping BAC instillation. CIC was performed on the 7th day after stopping BAC instillation and the rabbits were sacrificed. The eyeballs of all animals were removed and fixed in 4% paraformaldehyde solution for 24 h. The eyeballs were divided into nasal and temporal halves from 12 o’clock to 6 o’clock and were either frozen or processed for paraffin embedding. Paraffin sections were used for hematoxylin and eosin (H&E) staining and frozen sections were used for immunofluorescence detection of mucin MUC5AC. The tears were stored in the − 80℃ refrigerator, and the concentration of MMP-9 was detected by ELISA kit.

### Observation indicators and scoring criteria for dry eye examination

1. Scoring criteria for conjunctival congestion [16].

0 point: no congestion; 1 point: increased number of vessels, no obvious dilation; 2 points: increased number of vessels with obvious dilation; 3 points: blood vessels are dark red and distorted.

2) Corneal fluorescein sodium staining method and scoring criteria [16].

According to the depth of corneal staining (S), it is divided into: 0 – no corneal staining; 1 – only dot staining (mild); 2 – both slight and slice staining (moderate); 3 – continuous dark sheet staining (severe). The proportion of corneal area to the total area as A percentage of different degrees of staining was calculated as A%, S and A were multiplied, and the scores were added to the total score of corneal sodium fluorescein staining.

3) Corneal neovascular score [16].

The cornea was divided into four quadrants, each 0–4, for a total of 16. 0 point: no new blood vessels into the cornea; 1 point: only new blood vessels within 5 roots, new blood vessels are very short and thin; 2 points: 5–10 new blood vessels from 1 point; 3 points: new blood vessels with 10–20 new blood vessels; 4 points: new blood vessels with more than 20 dense blood vessels, difficult to count the roots. Total corneal neovascularization is the sum of the four quadrant scores.

4) Corneal edema score [16].

Edema degree: 0 point: no corneal edema, iris vessels clearly visible; 1 point: corneal edema, corresponding area iris vessels not clear; 2 points: corneal edema, corresponding area iris vessels blurred; 3 points: corneal edema, corresponding area iris vessels not visible; 4 points: corneal edema, corresponding area iris invisible and edema corneal bulge, uneven surface.

Edema range: 0: 0 to 1/4 corneal edema; 1: 1/4 to 1/2 corneal edema; 2: 1/2 to 3/4 corneal edema; 3: more than 3/4 corneal edema.

Total score of corneal edema = degree of edema score + edema range score.

5) Tiger red staining and scoring criteria.

The cornea was divided into four peripheral corneal areas and five areas of the pupil central corneal area, plus two nasal and temporal spherical conjunctival areas, and each area was scored 0–3 points, for a total of 21 points. 0: no dots staining; 1: 1–10 dots staining; 2: 11–30 dots staining; 3: >30 dots staining or fused clusters. Tiger red staining is the sum of the above seven regions.

### Histopathology

Twenty-four hours after fixation, the eyeball was cut in half into two parts along 6 o’ clock and 12 o’ clock through the optic nerve. The temporal eyeballs were placed in 4% paraformaldehyde solution, fixed in a 4 ℃ refrigerator for 24 h, then dehydrated and embedded in paraffin. Paraffin sections were 5 μm thick, dried for H&E staining after sectioning, and photographed for observation under a light microscope. The nasal eyeballs were transferred to 30% sucrose solution for dehydration overnight, embedded with OCT and placed in liquid nitrogen for coagulation then stored at − 80 °C or sectioned at a thickness of 8 μm.

### Immunofluorescent staining for MUC5AC

All frozen section samples were removed from the refrigerator at − 80 ℃, rewarmed at room temperature for 30 min, and then fixed in acetone at 4 ℃ for 10 min. The samples were washed with PBS solution for three times, 5 min each time, and then blocked with 10% goat serum (diluted in PBS solution) for 30 min at room temperature. The samples were incubated with mouse anti-rabbit MUC5AC antibody diluted 1:100 at 4 ℃ for 12 h. The samples were washed three times with PBS solution for 5 min each time, and the samples were incubated with FITC-labeled goat anti-mouse IgG for 45 min at room temperature, taking care to avoid light. The cells were washed three times with PBS solution, and 0.5 µg/mL Hoechst 33,342 dye was used for nuclear staining. The samples were observed and photographed using a confocal microscope.

### Statistical analyses

The data of clinical observations such as conjunctival hyperemia, corneal edema, corneal neovascularization, corneal fluorescein sodium staining and Rose Bengal staining in this study were graded repeated measures data. The generalized regression analysis of graded data was used, and other variables in the experiment were corrected and evaluated. For goblet cell counts, generalized multiple linear regression analysis was used with continuous normal distribution. Experimental data are presented as mean ± standard error for continuous data and as the count distribution of ranks. A *P* value of less than 0.05 was considered statistically significant. Statistical software JMP 15 (100 SAS Campus Drive, Cary, NC 27,513 − 2414, USA) was used.

## Results

### Measurement of drug membrane thickness and drug load measurement

**Fig. 2:**
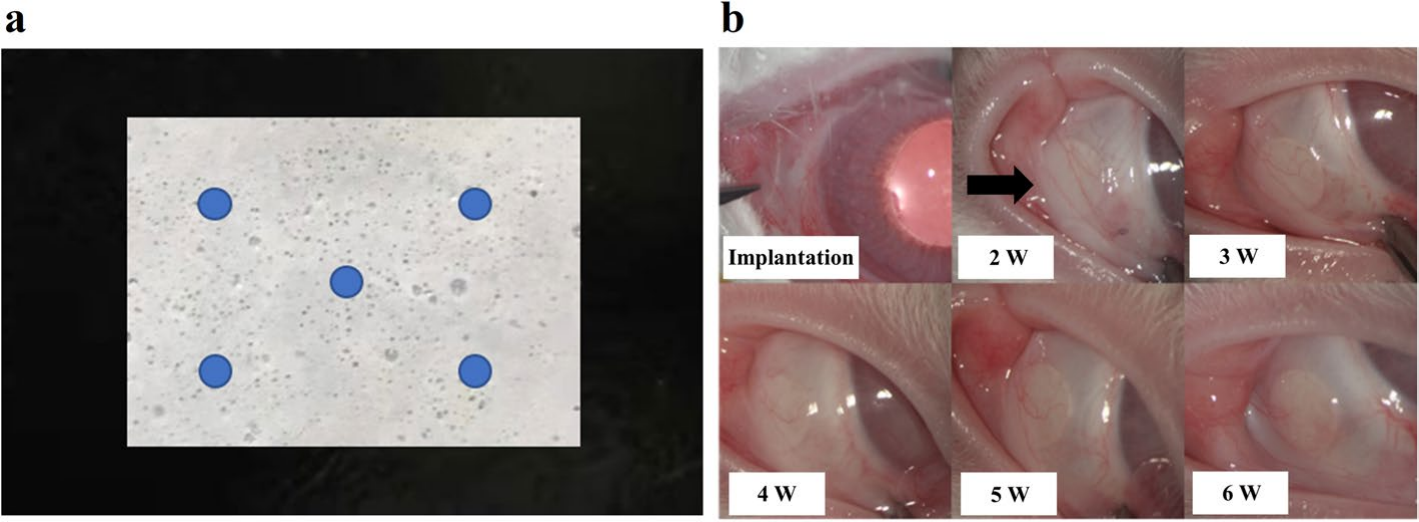
The images of drug membrane and its in vivo implantation. **a** Location where measurements for drug membrane thickness and drug load determination were taken. **b** The global conjunctival congestion around the implant was observed at the time of implantation and 2, 3, 4, 5, and 6 weeks after implantation (black arrow refers to the implant site). W, week

Five positions above the membrane (Fig. 2a) were selected for measuring the actual thickness of the membrane between 160 and 170 μm; the average thickness was found to be 165 ± 3.54 μm. By UPLC, the average load of the Lifitegrast is 30.09 ± 0.48%, and CsA is 28.1 ± 0.22%, which is almost consistent with the 30% of the two drugs designed with the membrane.

### Safety evaluation of the drug membrane

**Fig. 3:**
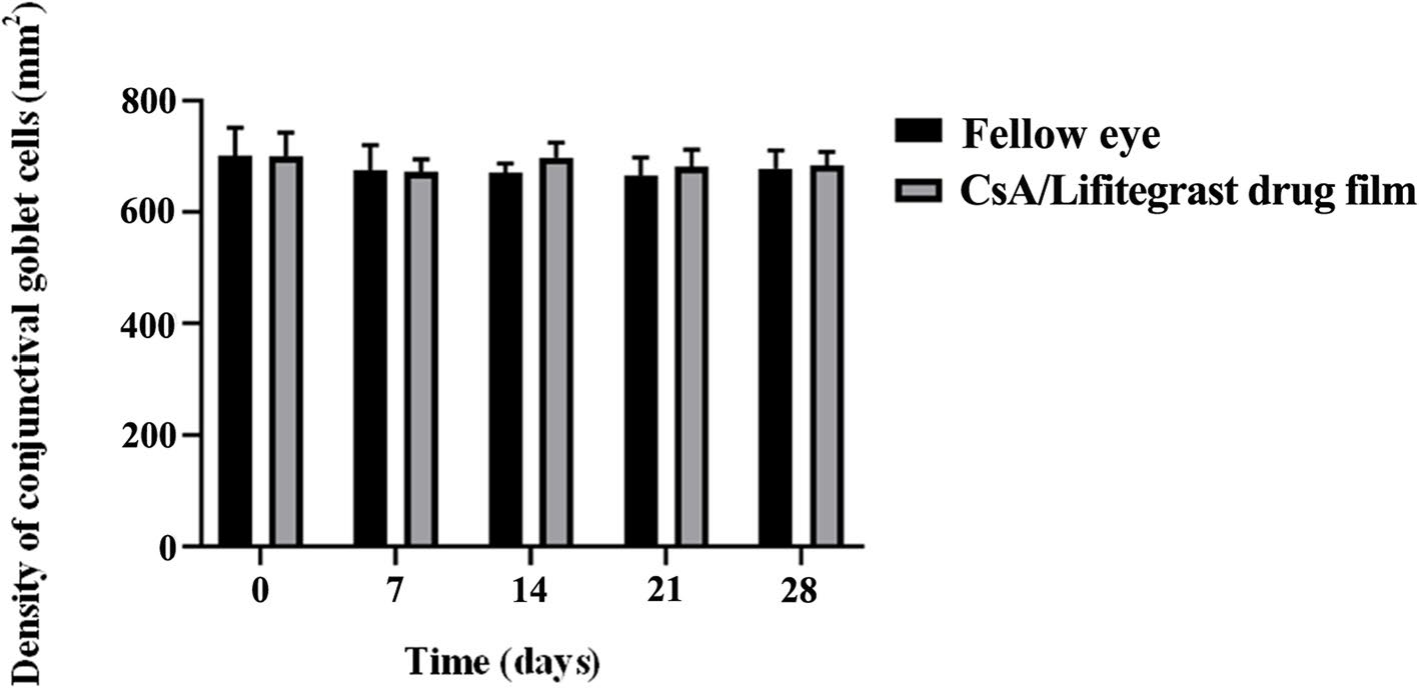
Density counts of conjunctival goblet cells at each observation time point of the implanted CsA/Lifitegrast composite membrane. Fellow eye represents left eye without implanted membrane, and CsA/Lifitegrast drug film represents right eye with implanted membrane

**Fig. 4:**
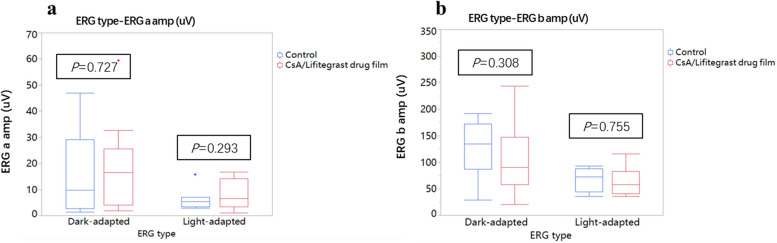
Electroretinogram (ERG) results. **a** Statistical results of ERG a-wave at 4 weeks of membrane implantation. **b** Statistical results of ERG b-wave at 4 weeks of membrane implantation. Control represents left control eye without membrane implantation. CsA/Lifitegrast drug film represents eye with membrane implantation

**Fig. 5:**
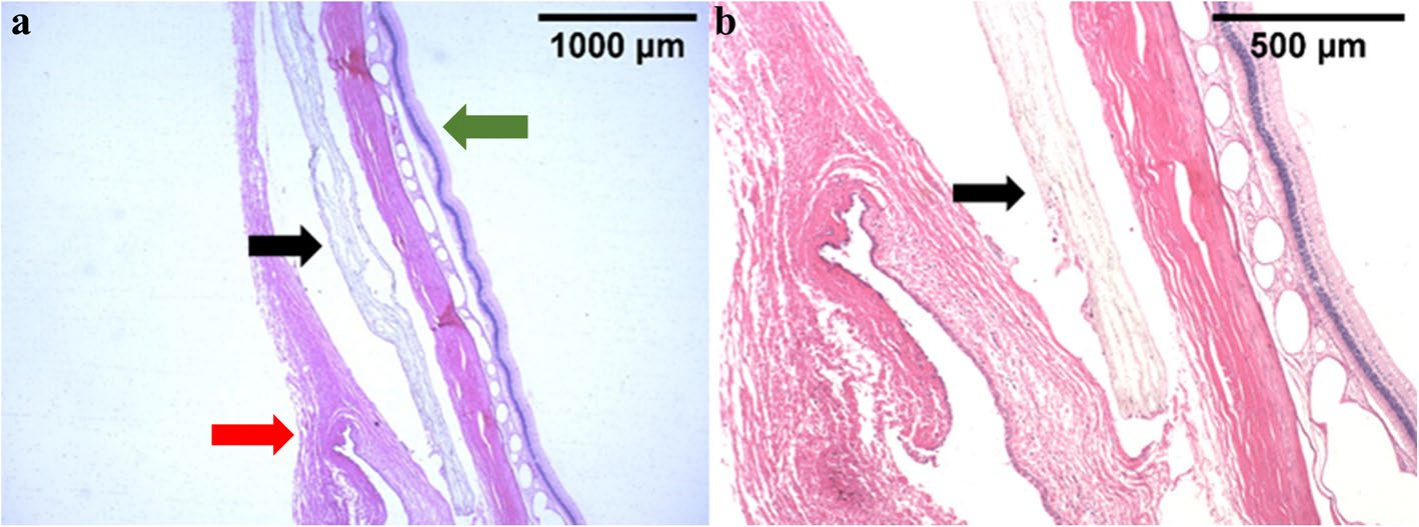
Pathological results of conjunctival tissue. Pathological section at (**a**) 10× magnification and (**b**) 20×magnification showing CsA/Lifitegrast composite membrane implanted under the conjunctiva of rabbit eyes after 6 weeks. Black arrow: membrane; red arrow: curled conjunctiva; green arrow: retina; left scale bar = 1000 μm, right scale bar = 500 μm

After membrane implantation, the slit lamp observed that the conjunctiva had mild congestion on the third day after implantation, and the conjunctiva then returned to normal. During the one-month safety assessment, there was no aqueous flare, anterior segment reaction of anterior chamber cells, corneal staining, or Rose Bengal staining present in the corneas and conjunctiva. Two weeks after implantation, the peripheral conjunctiva showed no bioincompatible inflammatory reaction (Fig. 2b). The results of conjunctival goblet cell count (Fig. 3) showed that the number of conjunctival goblet cells at 1, 2, 3, and 4 weeks after drug implantation was not significantly different from that at baseline (i.e., on Day 0, before drug membrane implantation; *P* > 0.05). Furthermore, there was no significant difference between the left and right eyes, indicating that the implantation of drug membrane did not cause significant changes in the number of conjunctival goblet cells. Electroretinogram (ERG) examination was performed on the left and right eyes 4 weeks after the membrane implantation. There was no significant difference between the left and right eyes of a-wave and b-wave under bright and dark adaptation, indicating that the membrane implantation had no significant effect on retinal function (Fig. 4). Pathological sections (Fig. 5) showed that there was no inflammation in the conjunctival tissue around the drug film at 6 weeks after implantation, and normal tissues grew into the drug film and the drug film gradually degraded over time, demonstrating that the biocompatibility of the drug film was good.

### Drug-membrane characterization

**Fig. 6:**
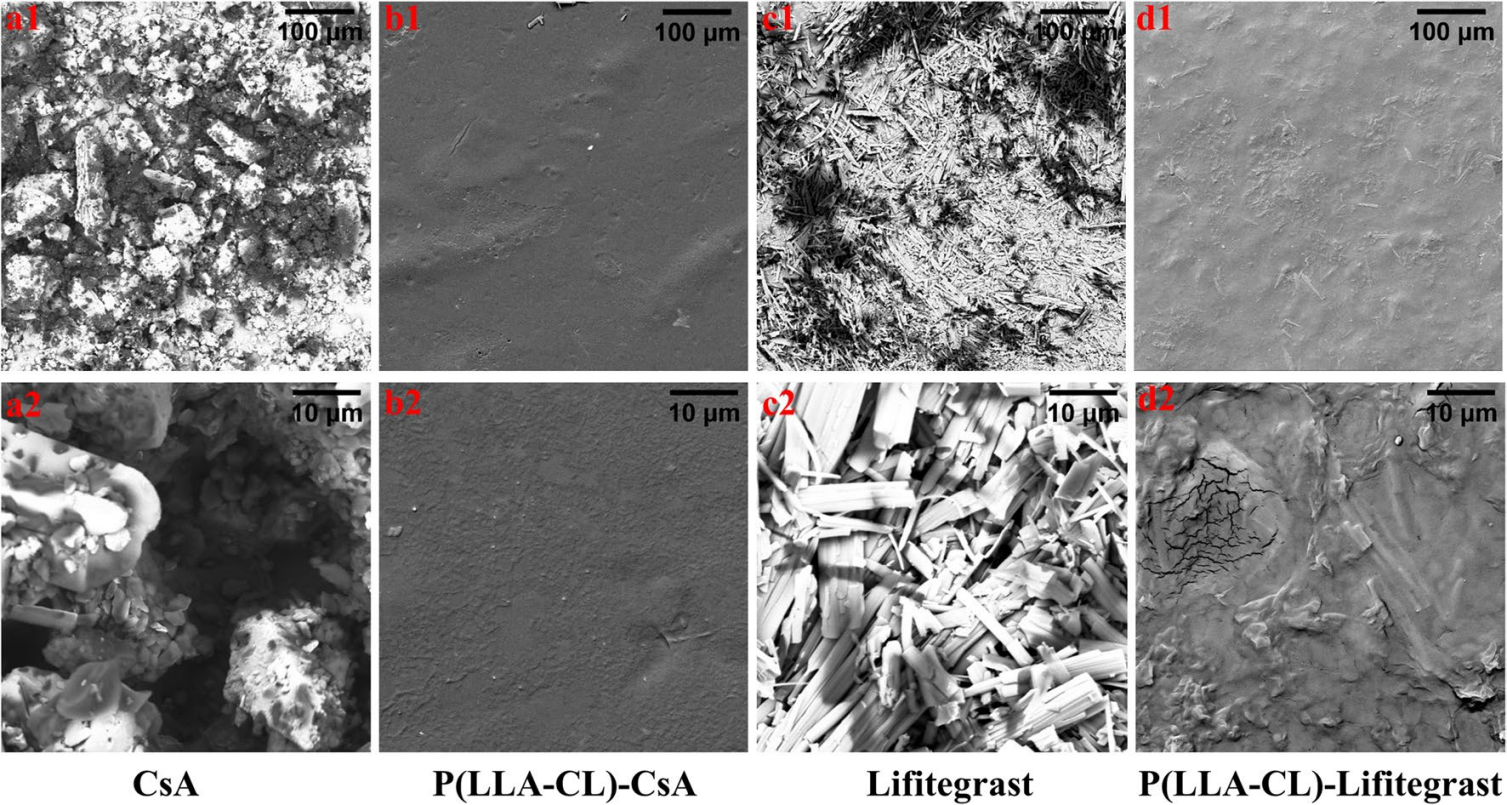
Scanning electron microscopy (SEM) results. **a1–a2** SEM images of CsA (naked drug); **b1–b2** SEM images of P(LLA-CL)-CsA (membrane CsA layer); **c1–c2** SEM images of Lifitegrast (naked drug); **d1–d2** SEM images of P(LLA-CL)-Lifitegrast (membrane Lifitegrast layer); top row: scale bar = 100 μm, and bottom row: scale bar = 10 μm

The drug-membrane composed of P(LLA-CL), CsA and Lifitegrast was prepared using a spray gun. Scanning electron microscopy (SEM) results (Fig. 6) showed that the naked CsA drug presented an irregular and rough mass-like structure, while the naked Lifitegrast drug presented a strip-like structure. Similar drug structures could be found in the CsA layer and Lifitegrast layer of the drug film, which confirmed the existence of the two drugs in the drug membrane.

**Fig. 7:**
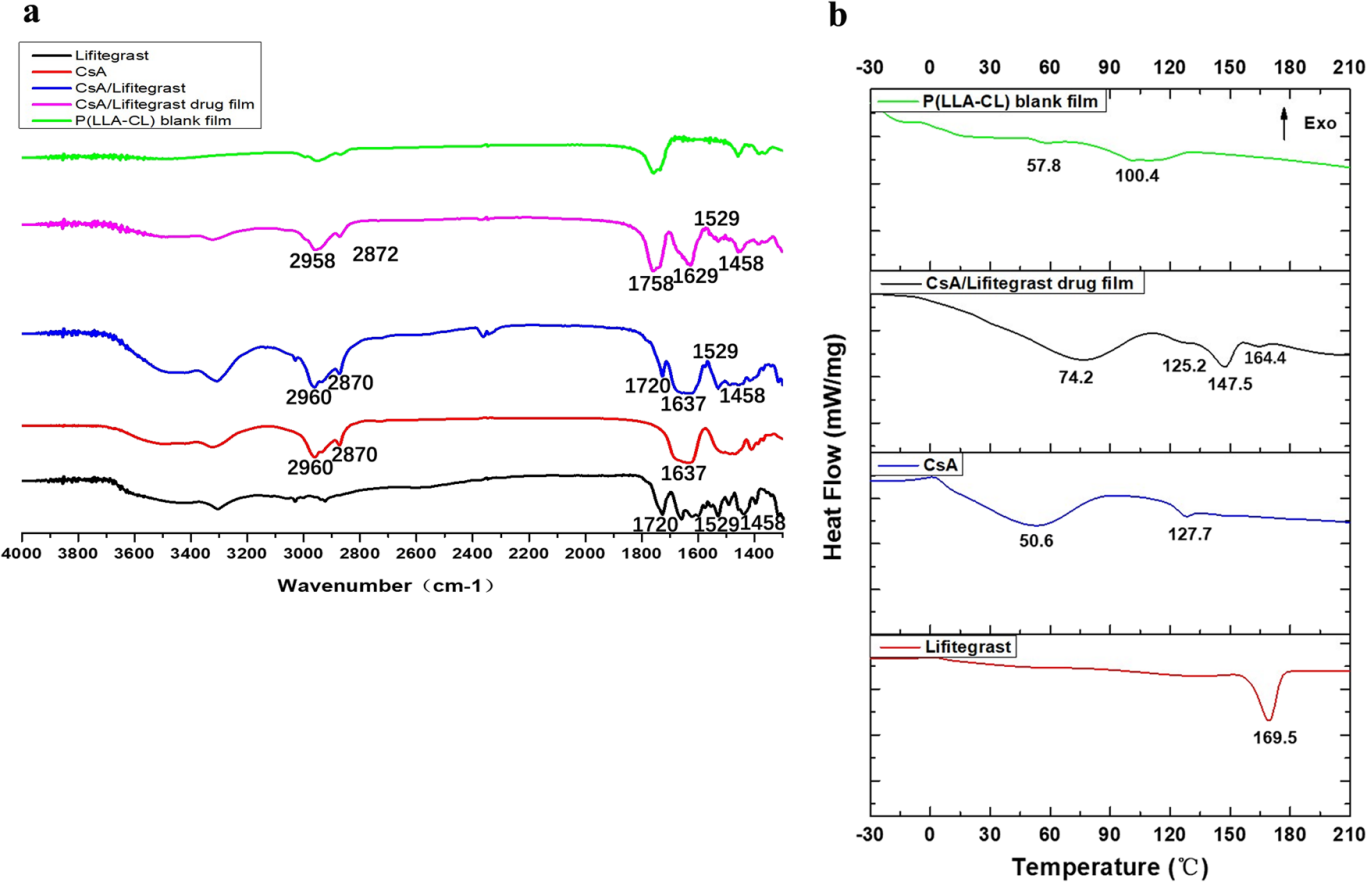
Fourier transform infrared spectroscopy (FTIR) and differential scanning calorimetry (DSC) results. **a** Infrared spectra of P(LLA-CL) blank film, CsA/Lifitegrast drug film, CsA/Lifitegrast (a mixture of two naked drugs), CsA (naked drug), and Lifitegrast (naked drug). **b** DSC profiles of CsA (naked drug), Lifitegrast (naked drug), P(LLA-CL) blank film, and CsA/Lifitegrast drug film

Fourier transform infrared spectroscopy (FTIR) results (Fig. 7a) showed that CsA had a typical infrared absorption band and the corresponding alkyl stretching vibration area at 2960 cm^−1^ and 2870 cm^−1^. There was a typical infrared absorption band at 1637 cm^−1^ which corresponded to the stretching vibration region of amide bonds. Lifitegrast showed a typical infrared absorption band at 1720 cm^−1^ which corresponded to the stretching vibration region of carbonyl functional group. A typical infrared absorption band was found at 1690–1620 cm^−1^ which corresponded to the stretching vibration region of amide bonds, and a typical infrared absorption band is found at 1600–1450 cm^−1^ which corresponds to the stretching vibration region of the C = C skeleton on the aromatic ring. The same infrared absorption band could be reproduced in the CsA/Lifitegrast mixed bare drug group. This indicates that no chemical changes occurred during the preparation of the drug film for the two drugs.

Differential scanning calorimetry (DSC) results (Fig. 7b) showed that the double endothermic peaks of CsA naked drug were 50.6 °C (starting point 3 °C) and 127.7 °C (starting point 120.9 °C), respectively. Lifitegrast bare drug has a single endothermic peak at 169.5 °C with a starting point of 160.4 °C. Four endothermic peaks appeared in the CsA/Lifitegrast composite drug film, respectively: 74.2 ℃ (starting point 8.9 ℃), 125.2 ℃ (starting point 116.8 ℃), 147.5 ℃ (starting point 138.6 ℃), 164.4 ℃ (starting point 158.6 ℃), which were similar to the peak shape of the two drugs, but because the drug content of the composite film was lower than that of the naked drugs, the peak height was smaller.

### In vitro release of drug membrane

**Fig. 8:**
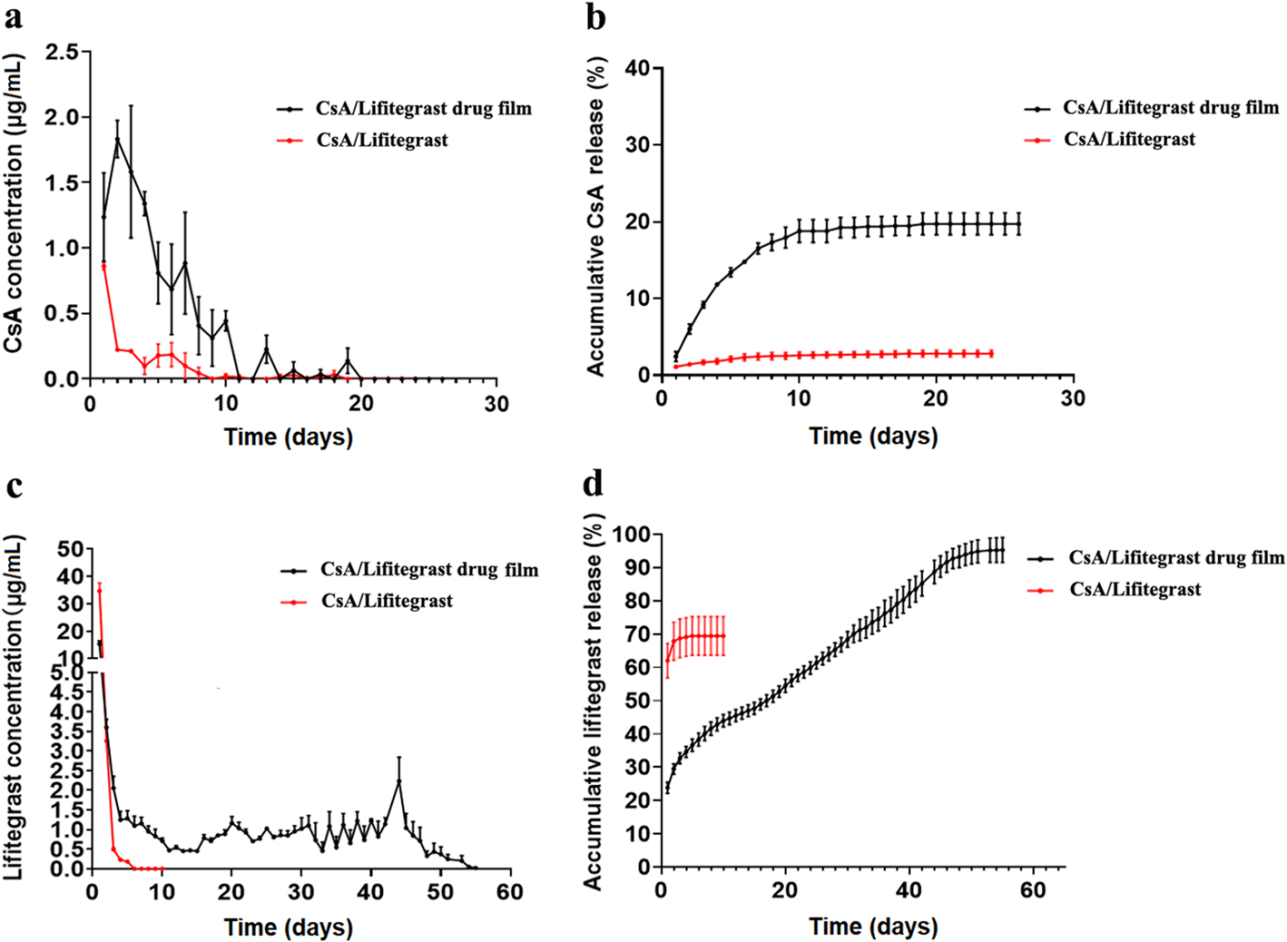
In vitro drug release test from the film. **a** Time-concentration profiles of CsA release in vitro. **b** In vitro time-cumulative release curve of CsA. **c** Lifitegrast in vitro release time-concentration curve. **d** Lifitegrast in vitro release time-cumulative release curve (black represents CsA/Lifitegrast composite membrane, red represents CsA/Lifitegrast mixed naked drug, dot is the mean, bar is the standard error

Figure 7 shows the in vitro release behavior of CsA/Lifitegrast composite film and CsA/Lifitegrast mixed naked drugs. The average content of CsA in CsA/Lifitegrast composite film was 253.29 ± 7.78 µg. The average amount of Lifitegrast was 322.39 ± 6.43 µg, the average amount of naked drug CsA used as a control was 250 µg, and the average amount of naked drug Lifitegrast used as a control was 320 µg. The results showed that the concentration of CsA in the composite drug film could still be detected on the 18th day after release in vitro, but the concentration of the naked drug group could not be detected on the 9th day after release in vitro. In the first 10 days of in vitro release, the concentration of CsA in the composite film was above 0.4 µg/mL, and the concentration of the naked drug was almost always lower than that of the drug film group which remained fairly constant at about 0.1 µg/mL. The cumulative release of the drug film group was about 20% of the total amount of CsA released, and that of the naked drug group was much lower than that of the drug film group at less than 5%. As for naked drug Lifitegrast, the average release concentration of Lifitegrast was 34.73 ± 5.0 µg/mL on the first day which then decreased to 3.25 ± 0.60 µg/mL on the second day. The cumulative release of Lifitegrast reached 70% of the total drug amount after 2 days. The release of Lifitegrast from the composite film in vitro also experienced a burst release on the first day, with an average release concentration of 15.39 ± 1.92 µg/mL, and an average release concentration of 1.02 ± 0.48 µg/mL on the 30th day which then maintained a relatively stable release rate. The release concentration remained essentially above 0.5 µg/mL until Day 54, with trace drug concentrations still detectable, and cumulative drug release approached 100% (Fig. 8). It can be seen from the above that the composite drug film significantly increased the release amount of Lifitegrast and achieved the purpose of sustained release. For CsA, although the synchronized sustained release effect of Lifitegrast was not achieved from the point of view of in vitro release, its release concentration and time were also increased.

### Pharmacodynamic study of the drug membrane

**Fig. 9:**
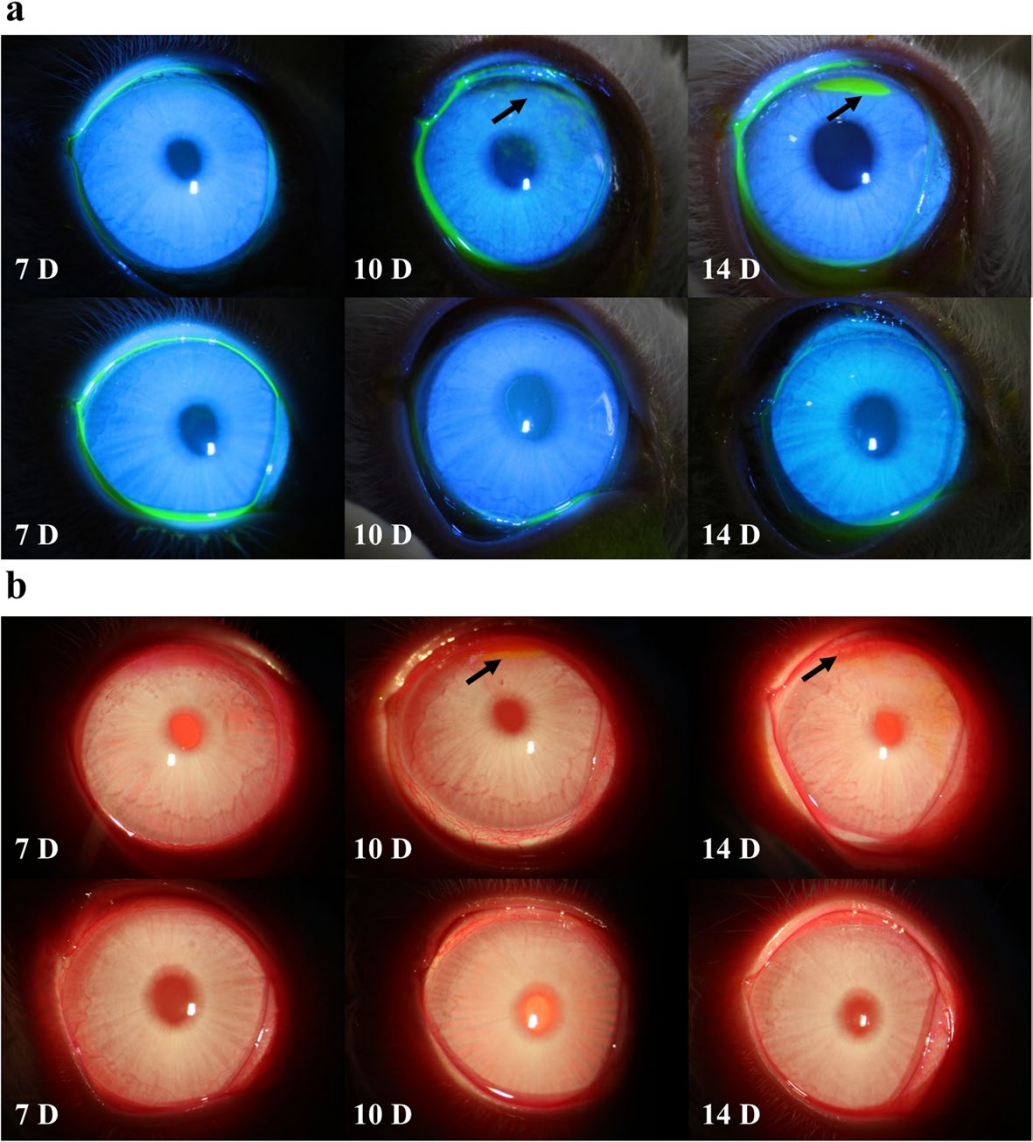
Observation of rabbit eyes using a slit lamp. **a** Pictures were taken under the fluorescein sodium staining slit lamp at each time point of the benzalkonium chloride (BAC) stage (top row: blank membrane group, bottom row: drug membrane group). **b** Tiger red staining and neovascular slit lamp at each time point of the BAC stage (top row: blank membrane group, bottom row: drug membrane group, arrow refers to the site of tiger red staining and neovascularization). D, days

**Fig. 10:**
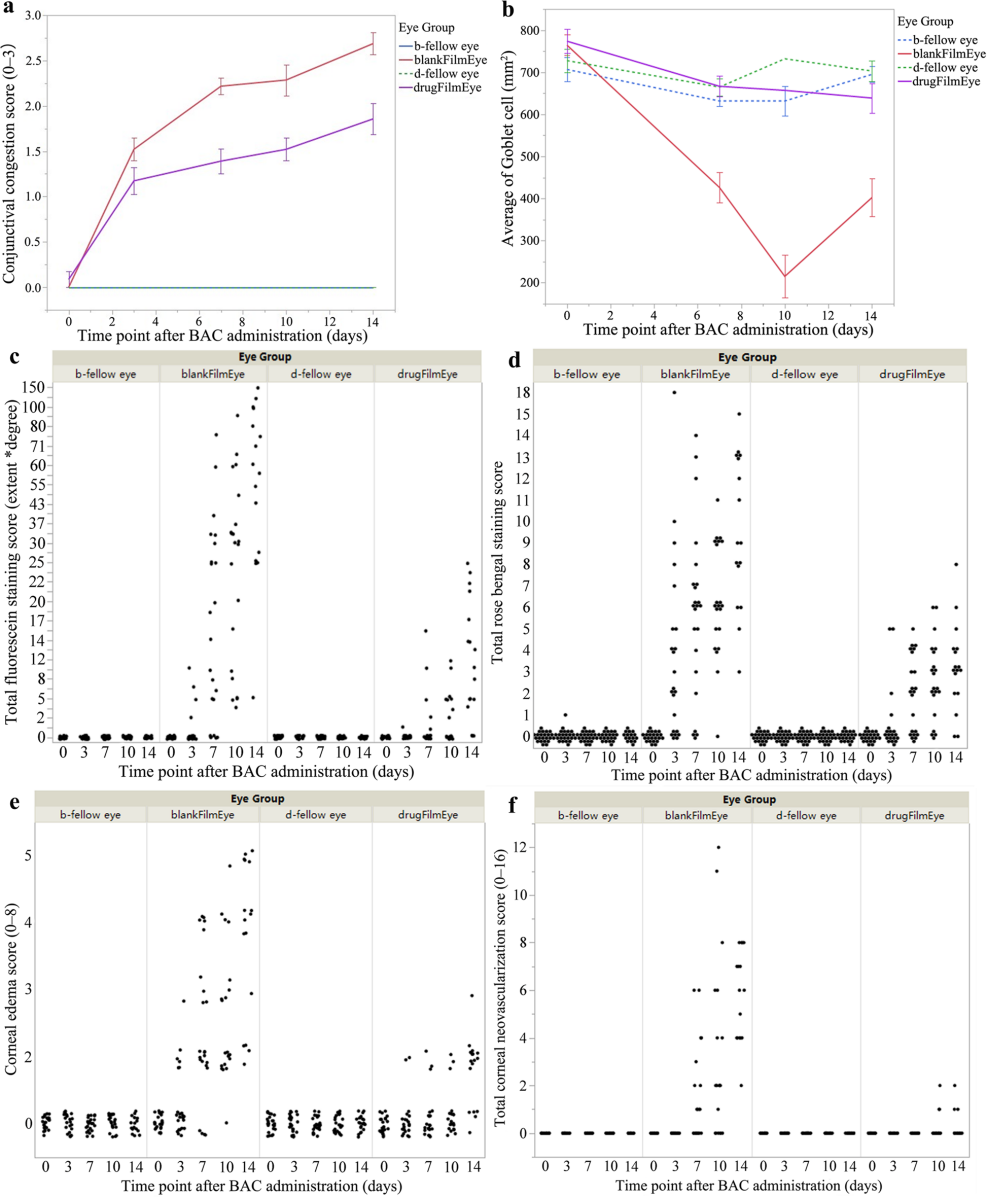
The treatment evaluation indicators of all groups. **a** Line chart of conjunctival congestion score; **b** Line chart of conjunctival goblet cell count; **c** Fluorescein sodium staining score, extent*degree: depth of corneal staining (S) multiplied with the proportion of corneal area to the total area (A%); **d** Rose bengal staining score; **e** Corneal edema score; **f** Distribution chart of corneal neovascularization score. b-fellow eye, the left eye treated without benzalkonium chloride (BAC) in the blank membrane group; blankFilmEye, the right eye treated with BAC in the blank membrane group; d-fellow eye, the left eye treated without BAC in the drug membrane group; drugFilmEye, the right eye treated with BAC in the drug membrane group

In this study, 46 Japanese white rabbits were used to induce dry eye models using BAC, with 23 animals in each group. In the period of dot BAC, it was observed that the drug membrane significantly inhibited conjunctival hyperemia score (drug membrane group median vs. blank membrane group median: 1 vs. 2, *P* < 0.001). Corneal fluorescein sodium staining score (median of drug membrane group vs. blank membrane group: 0 vs. 10, *P* < 0.001); Rose bengal staining score (median of drug membrane group vs. blank membrane group: 2 vs. 6, *P* < 0.001); corneal edema score (median of the drug membrane group vs. the blank membrane group: 0 vs. 1, *P* < 0.001) and corneal neovascularization score (median of the drug membrane group vs. the blank membrane group: 0 vs. 1, *P* < 0.001) (Figs. 9 and 10a, c, d, e and f). In addition, the results of conjunctival goblet cells (Fig. 10b) showed that the drug membrane inhibited the reduction of conjunctival goblet cells induced by BAC (mean ± SD: 653 ± 136 /mm^2^ vs. 398 ± 176 /mm^2^, *P* < 0.001). The recovery period of BAC in the two groups (12 animals in each group) showed that conjunctival hyperemia, corneal fluorescein sodium staining, corneal edema, corneal neovascularization and conjunctival goblet cells recovered faster in the drug membrane group, and the difference was statistically significant.

**Fig. 11:**
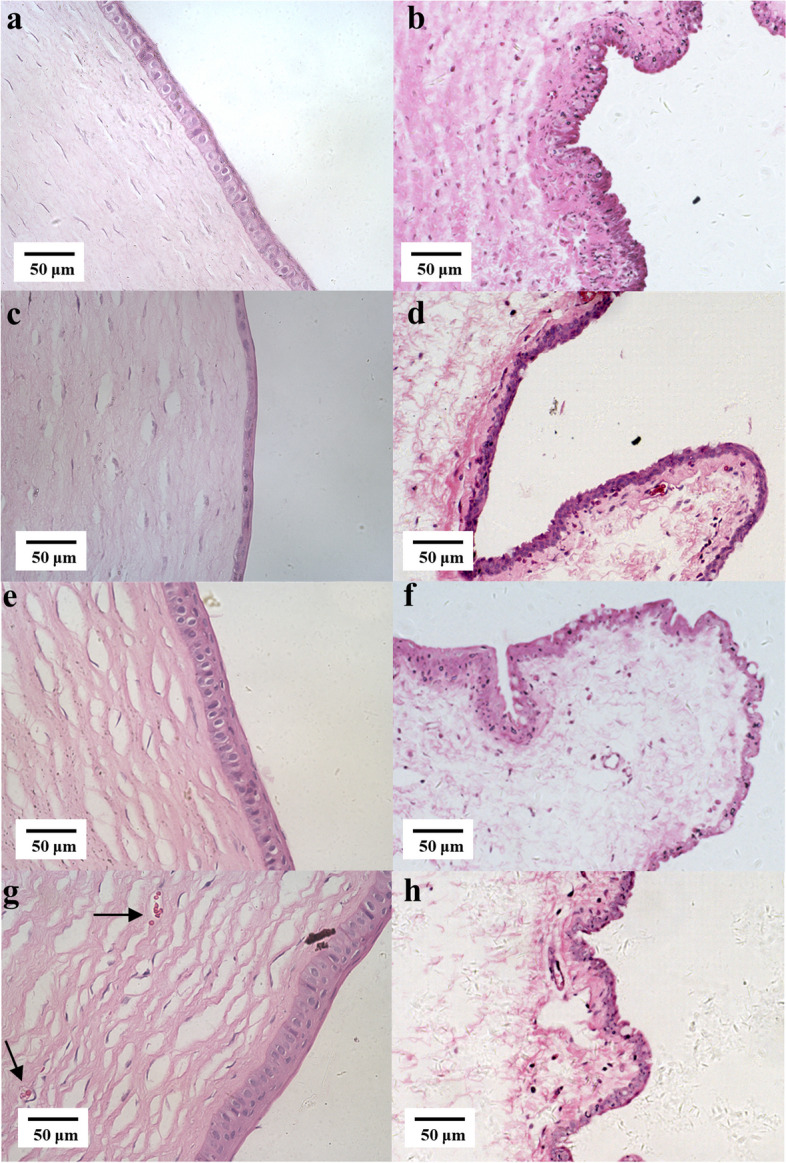
Hematoxylin and eosin (H&E) staining of the cornea and conjunctiva at different time points in benzalkonium chloride (BAC) treated eyes. **a**&**b** The cornea and conjunctiva of the drug membrane group; **c**&**d** The cornea and conjunctiva of the blank membrane group after 14-day BAC administration; **e**&**f** The cornea and conjunctiva of the drug membrane group after withdrawal of BAC for 7 days; **g**&**h** The cornea and conjunctiva of the blank membrane group after withdrawal of BAC for 7 days; black arrow indicates neovascularization, scale bar = 50 μm

**Fig. 12:**
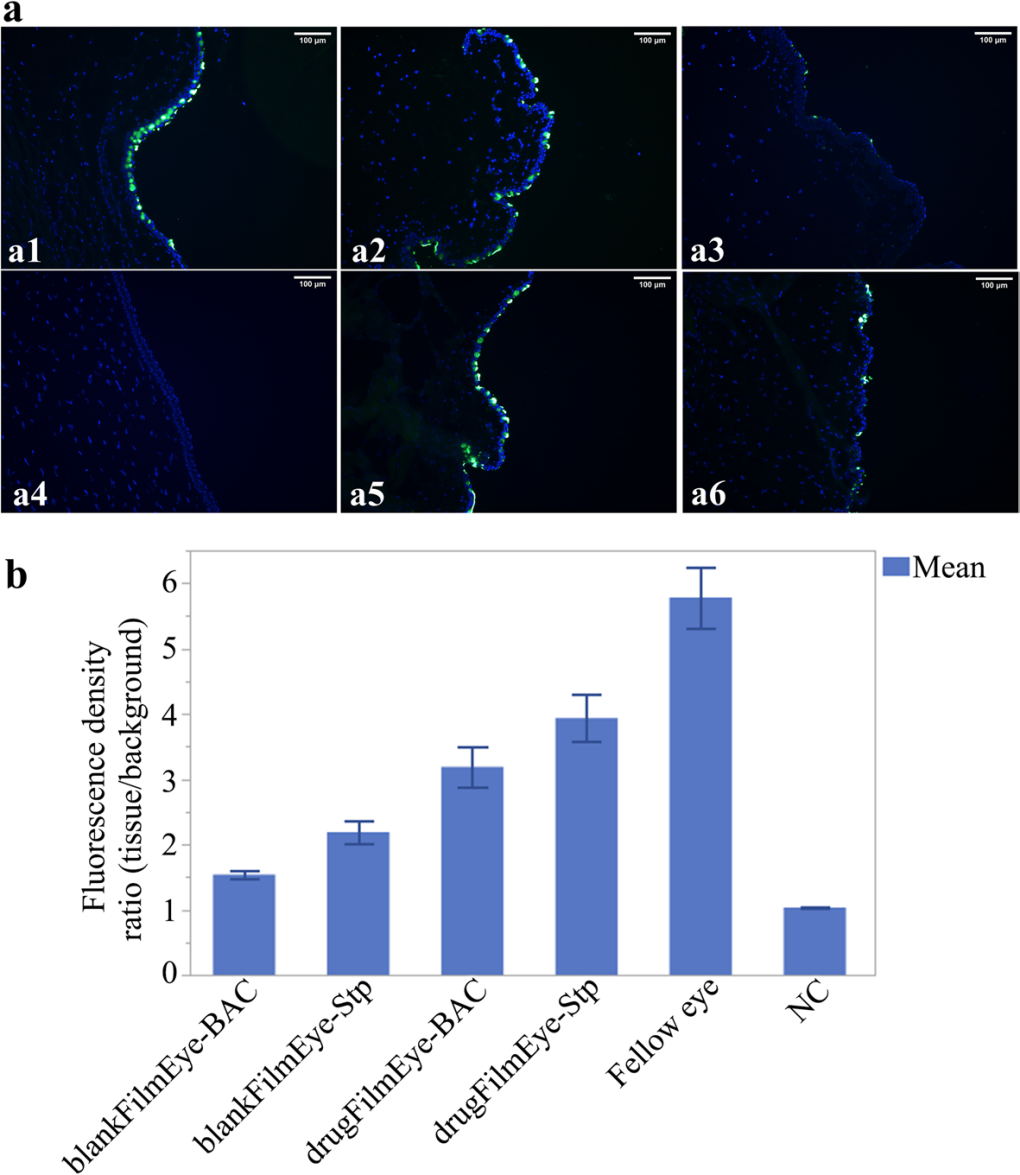
Immunofluorescence detection of mucin MUC5AC. **a** Immunofluorescent image of MUC5AC expression in conjunctival tissue. **a**1: Left eye without benzalkonium chloride (BAC); **a**2: Membrane implanted eye on day BAC 14; **a**3: Blank membrane implanted eye on day BAC 14; **a**4: Negative control i.e., corneal tissue; **a**5: Implanted eye 7 days after BAC withdrawal; **a**6: Blank membrane implanted eye 7 days after BAC withdrawal, scale bar = 100 μm). **b** Fluorescence density ratio of MUC5AC in the conjunctival epithelium in different eyes. blankFilmEye BAC, the eyes of blank membrane group with BAC 14 days; blankFilmEye-Stp, the eyes of the blank membrane group with BAC that was stopped for 7 days; drugFilmEye-BAC, the eyes of drug membrane group with BAC 14 days; drugFilmEye-Stp, the eyes of the drug membrane group with BAC that was stopped for 7 days; NC, the corneal tissue; Fellow eye, left eyes, i.e., control eyes not treated with BAC, Bar indicates standard error

**Fig. 13:**
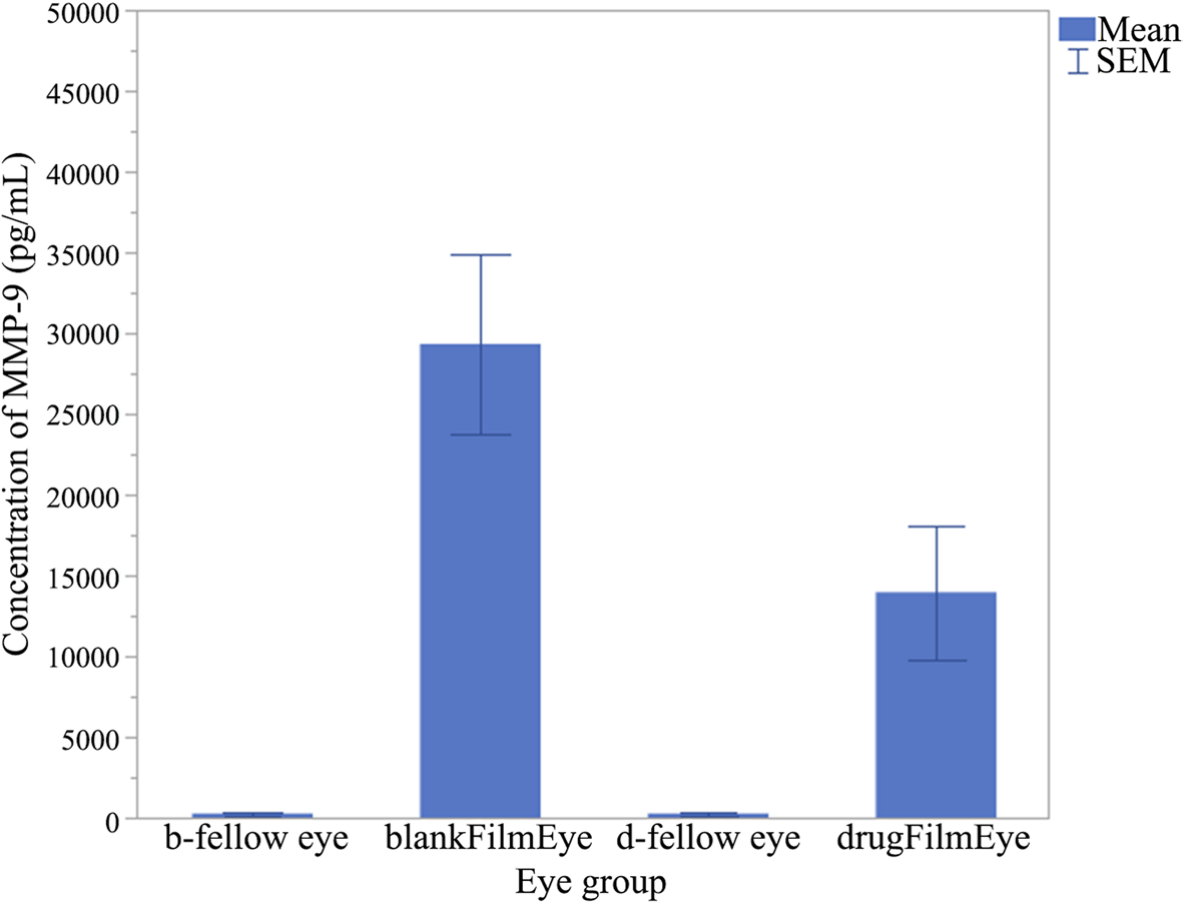
MMP-9 concentration in different groups. b-fellow eye, the left eye treated without BAC in the blank membrane group; blankFilmEye, the right eye treated with BAC in the blank membrane group; d-fellow eye, the left eye treated without BAC in the drug membrane group; drugFilmEye, the right eye treated with BAC in the drug membrane group. SEM, standard error of the mean

The corneal epithelium of the drug membrane group was significantly thicker, and there was no stromal edema and many apoptotic cells in the conjunctival epithelium (Fig. 11, a and b). At the same time, the blank membrane group had a significant reduction in the number of corneal epithelial cells, narrowing of the shape, edema of the corneal stroma, a large number of apoptotic cells with condensed nuclei in the conjunctival epithelium (Fig. 11, c and d). After 7 days of BAC-withdrawal, the number of apoptotic cells in the corneal epithelium, corneal neovascularization and goblet cells in the conjunctiva of the drug membrane group (Fig. 11, e and f) were still higher than those of the blank membrane group (Fig. 11, g and h). Immunofluorescence results (Fig. 12a1–a6) showed that the mucin MUC5AC in the drug membrane group was significantly higher than that in the blank membrane group, and the fluorescence density was counted by Image J (Fig. 12b). The detection results of MMP-9 in tears (Fig. 13) showed that the drug film inhibited the increase of this inflammatory factor (drug film group vs. blank film group: 13,967 ± 4154 pg/mL vs. 29,322 ± 5560 pg/mL, *P* < 0.001), which indicated that the drug film inhibited the reduction of mucin and the inflammatory response of the cornea and conjunctiva.

## Discussion

Dry eye is a malignant inflammatory cycle disease involving the activation of various inflammatory pathways and the release of inflammatory factors [4]. While artificial tears are available to alleviate dry eye symptoms, they only provide surface lubrication, fail to address the root cause, and cannot relieve severe cases. In fact, prolonged use of artificial tears can even cause corneal epithelial defects that develop into corneal ulcer, thinning and perforation. CsA [6] and Lifitegrast [7] are used to treat refractory dry eyes, but they are expensive and require four and two daily applications, respectively. Eye drops have a high clearance rate, which makes it challenging to maintain a consistent drug treatment concentration. As a result, the drug concentration is often too high, leading to ocular surface discomfort. Additionally, some patients find it inconvenient to administer multiple doses per day, which results in poor compliance. Hydrogels are also one of the commonly used carrier forms for ophthalmic drugs, offering advantages such as convenience of use and prolonged drug retention on the ocular surface [17, 18]. Some hydrogel formulations have already been applied in clinical ophthalmology. However, compared to the drug-loaded membrane prepared in this study, hydrogel formulations still suffer from limitations such as limited drug loading capacity, restricted to ocular surface administration, and most critically, the inability to achieve sustained-release effects, necessitating frequent dosing. In contrast, drug-loaded membrane, due to their high drug loading capacity and controllable drug release rate, offer sustained-release effects, and thus present broad prospects for clinical applications. Nonetheless, drug-loaded membranes currently have drawbacks, such as requiring implantation through surgery and possessing a certain level of invasiveness. Therefore, researchers aim to develop a sustained-release implantation device for minimally invasive subconjunctival implantation, specifically for patients with severe dry eyes. This implantation device would eliminate the need for multiple doses per day and inhibit cell and humoral immunity simultaneously, reducing the amount of drugs used and avoiding drug waste. Furthermore, sustained release would prevent the side effects of excessive first doses caused by multiple eye drops, providing a stable and safe therapeutic concentration.

The CsA/Lifitegrast composite film was created using a polymer material called P(LLA-CL), which is easily degradable [10]. The film was designed to achieve sustained release of both drugs by increasing the daily release concentration of the drugs and the materials degraded. However, the in vitro release duration of the CsA in the sustained release film is shorter than that of Lifitegrast, and there was not even a 100% cumulative release due to CsA’s extreme hydrophobicity and low solubility in PBS solution. The safety assessment of the sustained release device showed good biocompatibility during long-term implantation although there was some conjunctival congestion within the first week after the operation. The drug-loaded micro membranes implant we have developed are implanted through a conjunctival incision, which may aggravate ocular surface inflammation. However, the purpose of this study is to test the physicochemical properties, drug release behavior, safety, and pharmacology of the controlled-release drug-loaded microfilm in vitro, as well as to conduct preliminary evaluations. In the future, for clinical applications and implantation methods, we can adopt more minimally invasive approaches and strategies to reduce the impact on the microenvironment of the ocular surface. To minimize damage and ensure faster recovery, a special injection device will be developed for a simpler and faster process of implantation. While the combined drug sustained-release membrane showed significant therapeutic effects in the dry eye model, further improvement of this sustained-release system is still needed to prolong drug release time and coordinate sustained release-degradation time of the drug membrane.

Regarding whether the incision for surgical implantation would damage the opening of the lacrimal duct and affect the changes in tear secretion, we considered this issue before the surgery. Our experimental results indicate that the surgery does not cause damage to the opening of the lacrimal duct nor does it result in excessive damage to the conjunctival functional units. This is confirmed by the number of conjunctival cup cells before and after surgery, which showed no reduction postoperatively. Additionally, our incision size is small, located 3 mm from the corneal edge, and perpendicular to the corneal edge for the implantation of the drug film, ensuring that the farthest distance of the drug film does not exceed 8 mm. Furthermore, our lacrimal duct opening is located at the superotemporal conjunctival fornix, at least 8–10 mm away from the corneal edge. Finally, as mentioned earlier, we are developing a drug film injection device that requires no incision or sutures, allowing for faster and better drug film implantation while reducing damage.

Due to its simplicity in animal model preparation, BAC-induced dry eye model has been widely used by researchers. Clinical assessments indicate that local administration of BAC can elicit symptoms similar to human dry eye [19]. Therefore, this study employed a BAC-induced animal model of dry eye to evaluate the therapeutic effects and mechanisms of drug membrane. It can induce ocular surface inflammation, epithelial cell apoptosis, and squamous metaplasia, but cannot fully replicate the pathogenesis of immune-mediated dry eye [11]. For systemic disease-related dry eye, due to its complex etiology, modeling via eye drops is not feasible. In future studies, different dry eye models will be used to further confirm the effectiveness of the composite membrane. The core pathogenic mechanism of dry eye is immune inflammation, and the drugs we use are all intended for treating immune inflammation. These drugs exhibit effective therapeutic effects in various animal models, regardless of the specific type. Among these, MMP-9 is the most significant inflammatory marker of dry eye, elevated in severity. However, the pathogenesis of immune-related dry eye is complex and involves the interaction of multiple factors. In evaluating the therapeutic effects of CsA/Lifitegrast drug membrane, we only conducted MMP-9 detection. This inadequacy fails to fully explain these therapeutic effects, which is also a limitation of this study.

In addition, this study discovered that the rabbit dry eye model with 0.1% BAC eye drops exhibited different characteristics compared to the group with 0.2% BAC eye drops. Specifically, the length of the new vessels in the rabbit model was only half of the corneal radius and the density was as much as a brush, which required a more appropriate neovascularization scoring criterion. However, once BAC was stopped, the inflammatory symptoms quickly recovered, although certain inflammatory observation indicators persisted on the 7th day. Based on these findings, the 0.1% BAC dry eye model may not be suitable for long-term pharmacodynamic evaluation, but rather for testing drug effects within one week of stopping BAC. Reducing the BAC concentration or times of BAC eye drops maybe can create a more appropriate dry eye model for long-term drug evaluation.

## Conclusions

Here, we created a safe and effective CsA/Lifitegrast loaded P(LLA-CL) membrane which can be administered subconjunctivally. This can be developed into a minimally invasive delivery system to help patients with dry eye disease who require multiple daily eye drops but have poor compliance.

## Data Availability

The datasets used and analyzed during the current study are available from the corresponding author on reasonable request.
